# Caregiver Burden and Support Needs in Families of Patients With Parkinson’s Disease: A Mixed-Methods Investigation Combining Caregiver Strain Questionnaires and Focus Group Discussions

**DOI:** 10.7759/cureus.114338

**Published:** 2026-08-11

**Authors:** Misbah Shaikh, John Hippon, Neelima R Saoji, Mohit Agrawal

**Affiliations:** 1 Accident and Emergency, Ruby Hall Clinic, Wanowrie, Pune, IND; 2 Gastroenterology, KIMSHEALTH Trivandrum, Trivandrum, IND; 3 Medicine, N. K. P. Salve Institute of Medical Sciences and Research Centre and Lata Mangeshkar Hospital, Nagpur, IND; 4 Physical Medicine and Rehabilitation, S. S. Agrawal Institute of Physiotherapy and Medical Care Education, Navsari, IND

**Keywords:** caregiver burden, caregiver strain index, family caregivers, focus group discussions, mixed-methods study, parkinson’s disease, zarit burden interview

## Abstract

Background

Parkinson's disease is a progressive neurodegenerative disorder associated with declining functional independence and reduced quality of life. Family caregivers of individuals with Parkinson's disease frequently experience substantial emotional, physical, social, and financial challenges that may adversely affect their well-being and their ability to provide long-term care. This study evaluated caregiver burden and support needs using a mixed-methods approach that incorporated an investigator-developed caregiver burden questionnaire and focus group discussions.

Methods

This mixed-methods cross-sectional study included 102 family caregivers of individuals with Parkinson's disease attending a tertiary care neurology center. The quantitative component utilized an investigator-developed Caregiver Burden and Support Needs Questionnaire, developed following a review of published caregiver burden literature and common conceptual domains identified in caregiver burden research. Established caregiver burden instruments, including the Caregiver Strain Index (CSI) and Zarit Burden Interview (ZBI), were reviewed during questionnaire development; however, no copyrighted questionnaire items or scoring systems were reproduced or administered in this study. Qualitative data were collected through structured focus group discussions to explore caregivers' experiences, emotional challenges, and perceived support needs. Quantitative data were analyzed using descriptive and inferential statistics, while qualitative data were analyzed using thematic analysis.

Results

Among the 102 caregivers, 64 (62.8%) were female, and 58 (56.9%) were spouses of the care recipients. Overall, 81 (79.3%) participants experienced moderate-to-high caregiver burden. Higher caregiver burden was significantly associated with longer caregiving duration, greater daily caregiving hours, advanced disease stage, and cognitive impairment in the care recipient. Qualitative analysis identified four major themes: emotional distress, social isolation, financial burden, and unmet support needs. Participants frequently expressed the need for counselling services, respite care, caregiver support groups, and financial assistance.

Conclusion

Family caregivers of individuals with Parkinson's disease experience substantial emotional, physical, social, and financial burden, particularly when caregiving demands are prolonged or disease severity is greater. The integration of quantitative and qualitative findings highlights the need for comprehensive caregiver-centered psychosocial support services, including counselling, respite care, and structured support programs, to improve caregiver well-being and sustain long-term caregiving.

## Introduction

Parkinson's disease is a progressive neurodegenerative disorder characterized by a gradual decline in motor function accompanied by a range of non-motor symptoms that collectively impair an individual's ability to perform activities of daily living independently [1,2]. As the disease progresses, many individuals become increasingly dependent on family members for long-term care and assistance. Family caregivers often provide physical assistance, emotional support, supervision, medication management, and financial support, making them integral to the long-term management of Parkinson's disease. However, these caregiving responsibilities may impose substantial emotional, physical, social, and psychological burden on caregivers, adversely affecting their own health and well-being [3].

Several studies have identified multiple factors associated with increased caregiver burden among family caregivers of individuals with Parkinson's disease, including advanced disease severity, cognitive impairment, neuropsychiatric symptoms, and increasing functional dependence of the care recipient [2,4]. The prolonged demands of caregiving frequently result in psychological stress, fatigue, anxiety, social isolation, and financial strain as caregivers attempt to balance caregiving responsibilities with their personal, family, and occupational commitments [5]. Previous research has also demonstrated that female caregivers and spouses often experience greater caregiver burden because of prolonged caregiving duration and closer emotional involvement with the care recipient [6].

Standardized instruments, including the Caregiver Strain Index (CSI) and the Zarit Burden Interview (ZBI), are widely used to assess caregiver burden and psychological distress among family caregivers [7,8]. Although these instruments provide standardized quantitative measures, they may not fully capture caregivers' lived experiences, emotional challenges, practical difficulties, and perceived support needs. Consequently, qualitative approaches such as focus group discussions can provide a more comprehensive understanding of caregivers' experiences and their expectations from healthcare services [9].

A mixed-methods approach enables integration of quantitative and qualitative findings to provide a more comprehensive understanding of caregiver burden and support needs among family caregivers of individuals with Parkinson's disease [10]. The primary objective of this study was to determine the prevalence and severity of caregiver burden among family caregivers of individuals with Parkinson’s disease using an investigator-developed Caregiver Burden and Support Needs Questionnaire. The quantitative component also examined the associations of caregiver burden with relevant demographic, caregiving, and clinical factors, including caregiver sex, relationship to the care recipient, caregiving duration, daily caregiving hours, disease duration, disease stage, and cognitive impairment in the care recipient.

## Materials and methods

Study design and setting

A cross-sectional mixed-methods study was conducted in the Department of Neurology at a tertiary care academic institution to evaluate caregiver burden and support needs among family caregivers of individuals with Parkinson's disease using quantitative questionnaires and qualitative focus group discussions. Ethical approval was obtained from the Institutional Ethics Committee (Approval No. NKPSIMS & RC and LMH/IEC/20/2026) before commencement of the study. Written informed consent was obtained from all participants, and confidentiality was maintained throughout the study by assigning unique identification codes to each participant.

Selection Criteria

The inclusion criteria were family or non-family caregivers aged ≥18 years, providing care to an individual diagnosed with Parkinson's disease for at least six months, able to understand and respond to the questionnaire in English or the local language, and willing to provide written informed consent. The exclusion criteria were professional or paid caregivers, caregivers of individuals with atypical parkinsonism or other neurological disorders, individuals with documented psychiatric disorders, individuals with documented cognitive disorders, and those unwilling to participate.

Sample Size Calculation

The sample size was calculated using Cochran's formula, assuming a caregiver burden prevalence of 50%, a 95% confidence level, and a 10% margin of error. The minimum required sample size was 96 participants. To account for potential non-response, the target sample size was increased to 106 participants. Purposive sampling was used to recruit participants for the focus group discussions, and recruitment continued until thematic saturation was achieved.

Data sources and variables

Quantitative data were collected using an investigator-developed Caregiver Burden and Support Needs Questionnaire. The questionnaire was developed following a review of published literature on caregiver burden and support needs and common conceptual domains identified in caregiver burden research. Established caregiver burden instruments, including the CSI and ZBI, were reviewed during questionnaire development; however, no copyrighted questionnaire items or scoring systems were reproduced or administered in this study. The questionnaire assessed emotional burden, physical strain, social limitations, financial burden, caregiving demands, caregiver well-being, and perceived support needs (see Appendices).

Variables collected included caregiver age, sex, relationship to the patient, educational status, employment status, duration of caregiving, average daily caregiving hours, disease duration, disease stage, and the presence of cognitive impairment in the care recipient.

Qualitative data were collected through structured focus group discussions conducted by a trained moderator using a predefined discussion guide. Participants discussed caregiving experiences, emotional challenges, financial concerns, coping strategies, expectations from healthcare services, and perceived caregiver support needs. All focus group discussions were audio-recorded, transcribed verbatim, and analyzed using thematic analysis.

Development of the Caregiver Burden and Support Needs Questionnaire

The Caregiver Burden and Support Needs Questionnaire was developed by the investigators after reviewing published literature on caregiver burden in Parkinson’s disease and identifying commonly assessed conceptual domains, including emotional burden, physical strain, social limitations, financial burden, caregiving demands, caregiver well-being, and perceived support needs. Established instruments, including the CSI and ZBI, were reviewed to understand the principal domains commonly evaluated in caregiver research; however, no copyrighted items, wording, response options, scoring systems, or proprietary content were reproduced or administered.

The draft questionnaire was reviewed by the research team for clarity, relevance, comprehensibility, and alignment with the study objectives. However, formal face-validity testing, expert-based content-validity assessment, pilot testing, and psychometric evaluation were not conducted. Internal consistency was therefore not assessed using Cronbach’s alpha. The questionnaire findings should consequently be interpreted as descriptive results derived from an investigator-developed instrument rather than as scores obtained from a formally validated caregiver-burden scale.

Data Collection

Data were collected using paper-based questionnaires and structured focus group discussion guides administered by the investigators. Each participant was assigned a unique study identification number to maintain confidentiality. Completed questionnaires and consent forms were stored securely in a locked cabinet accessible only to the research team. Electronic data were entered into a password-protected database maintained on a secure institutional computer. Audio recordings and focus group transcripts were de-identified before analysis and stored in accordance with Institutional Ethics Committee requirements and institutional data confidentiality policies.

Statistical analysis

Quantitative data were entered into Microsoft Excel (Microsoft Corp., Redmond, WA, USA) and analyzed using IBM SPSS Statistics for Windows, Version 27 (Released 2019; IBM Corp., Armonk, New York, United States). Continuous variables are presented as mean ± standard deviation (SD), whereas categorical variables are expressed as frequencies and percentages. Independent-samples t-tests and one-way analysis of variance (ANOVA) were used to compare caregiver burden scores across participant demographic and caregiving characteristics. Pearson's correlation analysis was performed to evaluate the relationships between disease duration, daily caregiving hours, cognitive impairment, and caregiver burden scores. A two-tailed p-value of <0.05 was considered statistically significant. Qualitative data were analyzed using thematic analysis as described by Braun and Clarke [11]. Findings from the quantitative and qualitative analyses were integrated during interpretation to provide a comprehensive understanding of caregiver burden and support needs among family caregivers of individuals with Parkinson's disease.

## Results

Study demographics and caregiving characteristics are shown in Table 1. Caregivers generally have female gendered characteristics and are married to someone living with Parkinson’s disease. The average number of hours spent caregiving per day was approximately nine hours.

**Table 1:** Demographic and caregiving characteristics of caregivers (n = 102)

Parameter	Value
Mean age (years)	49.6 ± 12.4
Female	64 (62.8%)
Male	38 (37.2%)
Spouse caregivers	58 (56.9%)
Child caregivers	32 (31.4%)
Other relatives	12 (11.7%)
Employed	48 (47.0%)
Unemployed/housewife	54 (53.0%)
Mean daily caregiving hours	8.9 ± 3.5

Table 2 presents the clinical characteristics of participants and caregiver burden levels assessed using the investigator-developed questionnaire. The study showed that a significant number of caregivers experienced moderate-to-high levels of burden.

**Table 2:** Clinical characteristics and caregiver burden assessment (n = 102)

Parameter	Frequency (%)
Hoehn and Yahr Stage	
Stages I-II	40 (39.2)
Stage III	36 (35.2)
Stages IV-V	26 (25.6)
Cognitive impairment present	38 (37.2)
Caregiver burden category	
Low burden	21 (20.5)
Moderate burden	44 (43.1)
High burden	37 (36.2)

Caregiver burden scores are associated with variables related to caregiving, as shown in Table 3. Female caregivers, spouse caregivers, and those who provide at least eight hours of care daily tend to have higher burden scores.

**Table 3:** Association between caregiving variables and caregiver burden The independent t-test and the analysis of variance (ANOVA) were conducted to compare the mean caregiver burden scores of groups. All results had p-values < 0.05.

Variable	Mean caregiver burden score	p-value
Male caregivers	33.4	0.048
Female caregivers	36.2	
Spouse caregivers	38.1	0.041
Child caregivers	32.3	
Other relatives	30.7	
Caregiving hours <8/day	29.8	<0.001
Caregiving hours ≥8/day	40.5	

Table 4 displays the correlation of the clinical variables with the caregiver burden scores. Disease duration, cognitive impairment, and caregiving hours were statistically significant and positively correlated with the caregiver burden for each of the independent variables tested.

**Table 4:** Correlation between clinical variables and caregiver burden scores The Pearson correlation method has been employed here to determine if any correlation exists between caregiver burden scores and the clinical variables on which those scores are derived.

Variable compared	Correlation coefficient (r)	p-value
Disease duration vs caregiver burden score	0.52	<0.001
Daily caregiving hours vs caregiver burden score	0.48	<0.001
Cognitive impairment vs caregiver burden score	0.44	0.002

Table 5 outlines the key themes identified through focus group discussions as well as caregiver-reported areas of need. The common issues that arose among the caregivers were emotional distress, financial burdens, isolation from social support systems, and the need for respite care.

**Table 5:** Qualitative themes and caregiver support needs Themes were identified using thematic analysis according to Braun and Clarke’s framework.

Theme	Key findings
Emotional distress	Anxiety, burnout, frustration, guilt
Social limitations	Reduced social interaction and leisure activities
Financial burden	Medication expenses and reduced income
Support needs	Counselling, respite care, home-based rehabilitation
Preferred support services	Support groups and financial assistance

## Discussion

The present mixed-methods study evaluated caregiver burden and support needs among family caregivers of individuals with Parkinson's disease using an investigator-developed caregiver burden questionnaire together with focus group discussions. The findings demonstrated that a large proportion of caregivers experienced moderate-to-high caregiver burden, with emotional distress, social limitations, and financial strain emerging as the predominant challenges. The integration of quantitative and qualitative findings provided a comprehensive understanding of the multidimensional impact of caregiving for individuals with Parkinson's disease.

In the present study, most caregivers were middle-aged women and spouses of the care recipients. These findings are consistent with previous studies reporting that female family members, particularly spouses, frequently assume the primary caregiving role for individuals with Parkinson's disease [12,13]. Spousal caregivers often provide prolonged physical assistance, emotional support, and assistance with household responsibilities, all of which may contribute to increased caregiver stress and fatigue.

The investigator-developed caregiver burden questionnaire demonstrated that the majority of participants experienced moderate-to-high levels of caregiver burden, reflecting the substantial psychological, physical, and social demands associated with long-term caregiving. These findings are consistent with previous research demonstrating significant caregiver burden among family caregivers of individuals with neurodegenerative disorders [14,15]. Furthermore, caregiver burden increased significantly with longer caregiving duration, greater daily caregiving hours, and more advanced stages of Parkinson's disease, suggesting that increasing disease severity is associated with progressively greater caregiving demands.

Caregivers of individuals with cognitive impairment experienced significantly greater caregiver burden than those caring for cognitively intact individuals. Previous studies have similarly reported that cognitive impairment and neuropsychiatric symptoms substantially increase caregiver stress because of the additional supervision, behavioral management, and assistance with activities of daily living required by affected individuals [4,16]. These additional caregiving responsibilities may contribute to emotional exhaustion, anxiety, and reduced quality of life among caregivers. Prolonged caregiving may also affect health through neurobiological pathways associated with chronic stress. Repeated activation of the hypothalamic-pituitary-adrenal axis and sympathetic nervous system may alter cortisol regulation, autonomic balance, sleep, and metabolic function. Chronic stress may also reduce the sensitivity of immune-cell glucocorticoid receptors, weakening the normal anti-inflammatory effects of cortisol and promoting persistent low-grade inflammation. Studies of chronically stressed caregivers have demonstrated altered cortisol-to-dehydroepiandrosterone ratios, impaired hypothalamic-pituitary-adrenal feedback, and changes in cellular immune function. Other research has linked long-term psychological stress with oxidative stress, reduced telomerase activity, and accelerated telomere shortening, suggesting a potential pathway between sustained caregiving demands and premature cellular ageing. These mechanisms may partly explain the increased vulnerability of highly burdened caregivers to fatigue, sleep disturbance, depression, cardiovascular risk, and impaired immune health.

The qualitative findings complemented the quantitative results by highlighting caregivers' lived experiences of emotional exhaustion, social isolation, and financial strain. Many participants reported reduced participation in social activities, difficulty balancing caregiving responsibilities with employment and family commitments, and persistent emotional stress. Similar findings have been reported in previous qualitative studies exploring the experiences of family caregivers of individuals with Parkinson's disease [9,17].

Financial burden also emerged as a major concern. Participants described challenges related to medication expenses, long-term treatment costs, and reduced employment opportunities resulting from caregiving responsibilities. Caregivers additionally identified a need for counselling services, respite care, caregiver support groups, and rehabilitation services to assist them in managing the long-term demands of caregiving. Previous studies have shown that structured psychosocial support and family-centered interventions can improve coping strategies and reduce caregiver burden among families caring for individuals with chronic neurological disorders [5,6].

A major strength of the present study was the use of a mixed-methods design, which integrated quantitative assessment of caregiver burden with qualitative exploration of caregivers' experiences and support needs. The investigator-developed questionnaire, informed by published caregiver burden literature and common conceptual domains identified in caregiver burden research, enabled comprehensive evaluation of caregiver burden without relying solely on standardized quantitative measures. Focus group discussions further enhanced understanding by providing detailed insights into caregivers' emotional, social, and practical challenges. Collectively, these findings emphasize the importance of incorporating structured caregiver support services within comprehensive Parkinson's disease management programs.

Strengths and limitations

The present study has several strengths, including the use of a mixed-methods design that integrated quantitative assessment with qualitative exploration of caregiver experiences. The investigator-developed questionnaire, informed by published caregiver burden literature, together with focus group discussions, enabled a comprehensive evaluation of caregiver burden and support needs. However, several limitations should be acknowledged. The study was conducted at a single tertiary care center with a relatively modest sample size, which may limit the generalizability of the findings. In addition, caregiver burden was assessed using self-reported responses, making the results susceptible to recall and social desirability bias. Finally, the cross-sectional study design precludes assessment of changes in caregiver burden over time or establishment of causal relationships.

## Conclusions

Family caregivers of individuals with Parkinson's disease experience substantial emotional, physical, social, and financial burden, particularly when caregiving is prolonged, disease severity is advanced, and cognitive impairment is present in the care recipient. The mixed-methods approach employed in the present study demonstrated that caregiver burden extends beyond measurable caregiving demands to include significant unmet psychosocial and supportive care needs. The findings highlight the importance of integrating caregiver-centered interventions, including counselling services, respite care, caregiver support groups, and financial assistance, into comprehensive Parkinson's disease management programs to improve caregiver well-being and support the long-term sustainability of family caregiving.

## Appendices

Investigator-developed caregiver burden and support needs questionnaire

Development of the Questionnaire

The Caregiver Burden and Support Needs Questionnaire was independently developed by the investigators following a comprehensive review of the published literature on caregiver burden among family caregivers of individuals with Parkinson's disease and the conceptual domains commonly reported in caregiver burden research. During questionnaire development, established caregiver burden instruments, including the Caregiver Strain Index (CSI) and the Zarit Burden Interview (ZBI), were reviewed solely to identify commonly evaluated conceptual domains and to inform questionnaire design. However, no copyrighted questionnaire items, response options, scoring algorithms, proprietary content, or wording from these instruments were reproduced, adapted, or administered in the present study.

The final questionnaire was specifically designed for this study to assess emotional burden, physical strain, social limitations, financial burden, caregiving demands, caregiver well-being, and perceived support needs using original investigator-developed items. The questionnaire was reviewed and approved as part of the Institutional Ethics Committee-approved study protocol and was used exclusively for research purposes within the present study.

Instructions

Participants were asked to indicate the response that best reflected their caregiving experiences during the previous three months. Response scale: 1 = never, 2 = rarely, 3 = sometimes, 4 = often and 5 = always

**Table 6:** Caregiver burden and support needs questionnaire *Reverse scored items

Item No.	Question
1	I feel emotionally stressed because of my caregiving responsibilities.
2	Providing care leaves me physically exhausted.
3	I feel overwhelmed by the daily demands of caregiving.
4	My caregiving responsibilities limit my participation in social activities.
5	I have less time for my personal interests and hobbies because of caregiving.
6	Caring for my family member has created financial difficulties for me or my family.
7	The cost of medications, treatment, or related care places a burden on my household.
8	I spend a substantial amount of time each day providing care.
9	I worry frequently about the future health and care needs of my family member.
10	I find it difficult to balance caregiving responsibilities with work or household duties.
11	I feel anxious, frustrated, or emotionally drained because of caregiving.
12	I receive adequate emotional support from family members or friends.*
13	I am satisfied with my overall ability to cope with caregiving responsibilities.*
14	I would benefit from counselling or psychological support services.
15	I would benefit from respite care or temporary caregiving assistance.
16	I would benefit from caregiver support groups.
17	I would benefit from financial assistance or additional healthcare support services.
18	Overall, caregiving has negatively affected my quality of life.

Scoring and Interpretation

Each item was scored on a five-point Likert scale ranging from 1 to 5. Items 12 and 13 were reverse scored before calculation of total scores. Individual item scores were summed to generate a total score ranging from 18 to 90. Higher scores indicated greater caregiver burden and greater perceived support needs. Caregiver burden categories were low burden: total score < 36, moderate burden: total score 36-63, and high burden: total score > 63. Higher total scores reflected greater emotional strain, physical burden, social limitations, financial stress, caregiving demands, and unmet support needs among family caregivers of individuals with Parkinson’s disease.
